# Hepatic S6K1 Partially Regulates Lifespan of Mice with Mitochondrial Complex I Deficiency

**DOI:** 10.3389/fgene.2017.00113

**Published:** 2017-09-01

**Authors:** Takashi K. Ito, Chenhao Lu, Jacob Khan, Quy Nguyen, Heather Z. Huang, Dayae Kim, James Phillips, Jo Tan, Yenna Lee, Tuyet Nguyen, Samy Khessib, Natalie Lim, Surapat Mekvanich, Joshua Oh, Victor V. Pineda, Weirong Wang, Alessandro Bitto, Jonathan Y. An, John F. Morton, Mitsutoshi Setou, Warren C. Ladiges, Matt Kaeberlein

**Affiliations:** ^1^Department of Pathology, University of Washington Seattle, WA, United States; ^2^Department of Cellular and Molecular Anatomy, Hamamatsu University School of Medicine Hamamatsu, Japan; ^3^International Mass Imaging Center, Hamamatsu University School of Medicine Hamamatsu, Japan; ^4^Research Institute of Atherosclerotic Disease, Xi'an Jiaotong University Cardiovascular Research Center Xi'an, China; ^5^Laboratory Animal Center, Xi'an Jiaotong University Health Science Center Xi'an, China; ^6^Department of Comparative Medicine, University of Washington Seattle, WA, United States

**Keywords:** S6K1, mTORC1, liver, lifespan, mitochondrial disease

## Abstract

The inactivation of ribosomal protein S6 kinase 1 (S6K1) recapitulates aspects of caloric restriction and mTORC1 inhibition to achieve prolonged longevity in invertebrate and mouse models. In addition to delaying normative aging, inhibition of mTORC1 extends the shortened lifespan of yeast, fly, and mouse models with severe mitochondrial disease. Here we tested whether disruption of S6K1 can recapitulate the beneficial effects of mTORC1 inhibition in the Ndufs4 knockout (NKO) mouse model of Leigh Syndrome caused by Complex I deficiency. These NKO mice develop profound neurodegeneration resulting in brain lesions and death around 50–60 days of age. Our results show that liver-specific, as well as whole body, S6K1 deletion modestly prolongs survival and delays onset of neurological symptoms in NKO mice. In contrast, we observed no survival benefit in NKO mice specifically disrupted for S6K1 in neurons or adipocytes. Body weight was reduced in WT mice upon disruption of S6K1 in adipocytes or whole body, but not altered when S6K1 was disrupted only in neurons or liver. Taken together, these data indicate that decreased S6K1 activity in liver is sufficient to delay the neurological and survival defects caused by deficiency of Complex I and suggest that mTOR signaling can modulate mitochondrial disease and metabolism via cell non-autonomous mechanisms.

## Introduction

Reduced nutrient signaling, accomplished either by dietary restriction (DR) or genetic inhibition of the mTOR pathway, can robustly extend lifespan in many different species and protect against multiple age-related disorders (Johnson et al., 2013a). The mTOR kinase functions in two distinct complexes, mTOR complex 1 (mTORC1) and mTOR complex 2 (mTORC2), and plays an essential role in coordinating anabolic and catabolic processes in response to nutrients and growth factors (Laplante and Sabatini, 2013). Of the two TOR complexes, mTORC1 appears to play the most important role in normative aging, with numerous studies documenting lifespan and healthspan extension in mice following genetic knockdown of mTORC1 or treatment with the mTORC1 specific inhibitor rapamycin (Johnson et al., 2015a).

Ribosomal protein S6 kinase 1 (S6K1) is a substrate of mTORC1 that has been shown to modulate lifespan in invertebrates (Kapahi et al., 2004) and mice (Selman et al., 2009). Unlike rapamycin, which significantly increases lifespan in both male and female mice (Miller et al., 2014; Bitto et al., 2016), deletion of S6K1 extends lifespan specifically in female C57BL/6 mice, and the magnitude of this effect is less robust than that of rapamycin (Selman et al., 2009). A phosphorylation-defective allele of the S6K1 substrate glutamyl-prolyl-tRNA synthetase (EPRS) similarly extends lifespan in mice and recapitulates the low body weight of S6K1 knockout mice (Arif et al., 2017). These studies support a model that genetic or pharmacological inhibition of mTORC1 increases lifespan partially through reduced phosphorylation of S6K1 and its substrate EPRS.

Mitochondrial degeneration is one of the hallmarks of aging (Lopez-Otin et al., 2013), and declining mitochondrial function is implicated in a wide range of age-related disorders such as cancer, cardiovascular diseases, diabetes, sarcopenia, and neurodegenerative disorders (Payne and Chinnery, 2015; Rose et al., 2016; Kauppila et al., 2017; Sebastian et al., 2017). Genetic defects in nuclear or mitochondrial-encoded mitochondrial genes can lead to severe mitochondrial disorders such as Leigh Syndrome, MELAS (Mitochondrial encephalomyopathy, lactic acidosis, stroke-like episodes) syndrome, and Leber's hereditary optic neuropathy, which are often characterized by seizures, stroke, and progressive encephalopathy and myopathy (Lombes et al., 1989). Although, patients with severe mitochondrial disease do not show broad phenotypes of accelerated aging, normative aging may recapitulate some features of mitochondrial disease as a result of mitochondrial dysfunction. Consistent with this, both normative aging and severe mitochondrial dysfunction are associated with hyperactivation of mTOR signaling in multiple animal and cell-based models (Blagosklonny, 2006; Johnson et al., 2013b; Kim et al., 2013; Peng et al., 2015; Wang et al., 2016; Zheng et al., 2016). Several studies have indicated that genetic or pharmacological inhibition of mTOR can rescue defects associated with severe mitochondrial disease in yeast (Schleit et al., 2013), worms (Peng et al., 2015), fruit flies (Wang et al., 2016), and mice (Johnson et al., 2013b, 2015b).

The Ndufs4 knockout (NKO) mouse is a rodent model of human Leigh Syndrome, which develops progressive necrotizing encephalopathy of the vestibular nuclei, cerebellum, and olfactory bulb (Kruse et al., 2008; Quintana et al., 2010). Mice or humans lacking Ndufs4 have reduced Complex I levels and activity, and mutations in Ndufs4 cause Leigh Syndrome in humans (Ortigoza-Escobar et al., 2016). NKO mice are small but develop normally until about postnatal day 35 (P35) when they begin to display characteristic neurological phenotypes, progressive neuroinflammation and neurodegeneration, and brain lesions similar to those present in human Leigh Syndrome patients. NKO mice also show a profound decrease of body fat compared to their wild type (WT) or heterozygous littermates, and typically die between P50 and P60 (Johnson et al., 2013b).

We have previously reported that high dose rapamycin treatment is sufficient to delay mitochondrial disease in the NKO mice and suppress disease phenotypes including neurodegeneration, hyperactivation of mTOR, and low body fat (Johnson et al., 2013b, 2015b). To assess whether S6K1 plays a role downstream of mTOR in mediating mitochondrial disease progression, we crossed whole body and tissue-specific S6K1 knockout mice into the NKO background and examined the impact on health and survival. Here we report the observation that whole body disruption of S6K1 modestly rescues mitochondrial disease caused by loss of Ndufs4, but not to the same extent as rapamycin treatment. Unexpectedly, the magnitude of the rescue by whole body deletion of S6K1 can be achieved by disruption of S6K1 only in the liver, while disruption of S6K1 in brain or fat tissue had no effect on survival or disease progression in the NKO mice.

## Materials and methods

### Animals

Generation of S6K1 floxed mice (S6K1^fl/fl^) in C57Bl/6Ncrl strain and Cre-recombinase-expressing transgenic mice under the CMV promoter in C57Bl/6J strain was described previously (McQuary et al., 2016). *Ndufs4*^+/−^ breeders were obtained from the Palmiter laboratory at the University of Washington and backcrossed to C57Bl/6Ncrl strain as previously described (Johnson et al., 2013b). Cre-recombinase-expressing transgenic mice under the Albumin-promoter (JAX stock #003574), Adiponectin-promoter (JAX stock #010803), and Synapsin1-promoter (JAX stock #003966) were obtained from Jackson laboratory. *Ndufs4*^+/−^ mice and S6K1^fl/fl^ mice were bred to each other, and then crossed with each Cre-recombinase line, finally generating Cre; S6K1^fl/fl^; *Ndufs4*^+/−^ and S6K1^fl/fl^; *Ndufs4*^+/−^ (a hybrid of C57Bl/6Ncrl and C57Bl/6J) as breeders to produce *Ndufs4*^−/−^ offspring. Synapsin1-cre was always kept on the maternal side to avoid germline recombination caused by Synapsin1-cre line on the paternal side. Littermates were used in experiments so that all controls were appropriately genetically matched.

Mice were weaned at 3 weeks of age, except for cases where *Ndufs4*^−/−^ mice were too small, in which case these mice were not weaned until they reached body weights of at least 7.0 g. *Ndufs4*^−/−^ animals were housed with a minimum of one *Ndufs4*^+/+^ or *Ndufs4*^+/−^ littermate for warmth and stimulation. All care of experimental animals was in accordance with the institutional guidelines of University of Washington and Hamamatsu University School of Medicine, and experiments were performed as approved by the Institutional Animal Care and Use Committees at the University of Washington (protocol #4359-03) and Hamamatsu University School of Medicine (protocols H28-068 and H29-083).

### Genotyping PCR

Genotyping PCR to detect WT, NKO, Floxed, and Floxed-out alleles was performed using the following primers. 5′ primer S6K1 FO E 5′-GCTCAGCAGTTAAAGAGTACCGAC-3′, 5′ primer S6K1 WT F 5′-AGCCAGTATTGCAGTGCTTTGTGC-3′, and 3′ primer S6K1 WT F/FO 5′-TGGCACAGGTTGTTGCCACAATGA-3′. Primers for the floxed out signal (FO E and WT F/FO) are located upstream of the 5′ lox p site and downstream of the 3′ lox p site. Primers for the floxed and wildtype signals (WT F and WT F/FO) are located upstream and downstream of the 3′ lox p site. Primers used to detect Cre-recombinases were 5′-GTTCGCAAGAACCTGATGGACA-3′ and 5′-CTAGAGCCTGTTTTGCACGTTC-3′. Primers used to detect Ndufs4 KO and WT alleles were 5′-GGTGCATACTTATACTACTAGTAG-3′, 5′-AGCCTGTTCTCATACCTCGG-3′, and 5′-GCTCTCTATGAGGGTACAGAG-3′. The sizes of PCR products were designed to be 480 bp for WT, 580 bp for flox, and 656 bp for flox-out alleles.

### Lifespan

All mice were monitored daily, and sterile water gel was provided on the bottom of each cage. Mice were medicated for non-life-threatening conditions as directed by the veterinary staff. The dates of death were documented when mice died or were euthanized due to end-of-life criteria and were unlikely to survive longer than 48 h at the time of inspection. Mice were euthanized if they showed a loss of mobility, the ability to eat and drink, gait control and balance, righting reflex, or more than 30% of their maximum body weight.

### Western blotting

Tissues were flash-frozen in liquid nitrogen immediately after harvest and kept at −80°C until use. RIPA buffer (10 mM Tris-HCl pH 8.0, 1 mM EDTA, 1% Triton X-100, 0.1% sodium deoxycholate, 0.1% SDS, 140 mM NaCl, Roche Complete Ultra protease inhibitor, and PhosSTOP phosphatase inhibitor tablets) was added to the tube prior to homogenization with a grinder (Biorad). Protein lysates were quantified with a standard BCA assay. Twenty-five micrograms protein per sample were separated using SDS-PAGE and then transferred to a PVDF membrane for antibody probing. Antibodies used for S6K1 and Actin were purchased from Cell Signaling Technology.

### Clasping behavior

Forelimb clasping behavior was measured daily as a widely used sign of neurological degeneration as previously described (Kruse et al., 2008). Clasping involves an inward curling of the spine and a retraction of forelimbs or all limbs toward the midline of the body. Mice were picked up by the tail to judge if they showed the phenotype.

### Body fat ratio

The body composition of animals was analyzed in a non-invasive manner using quantitative magnetic resonance methods (Echo Medical Systems, Houston, TX). Non-anesthetized mice were placed in the sample holder and the sample holder was inserted into the center of the magnetic resonance machine. Each animal underwent 2 measurements unless the difference between measurements was over 5%, in which case a third measurement was performed. The body fat mass was calculated as the ratio of the average of the fat mass to the body weight of the animal.

### Statistical analysis

*p*-values for lifespan analysis were calculated using the Log-Rank test. Unpaired *t*-tests were used for other assays, unless otherwise noted.

## Results

**Figure 1:**
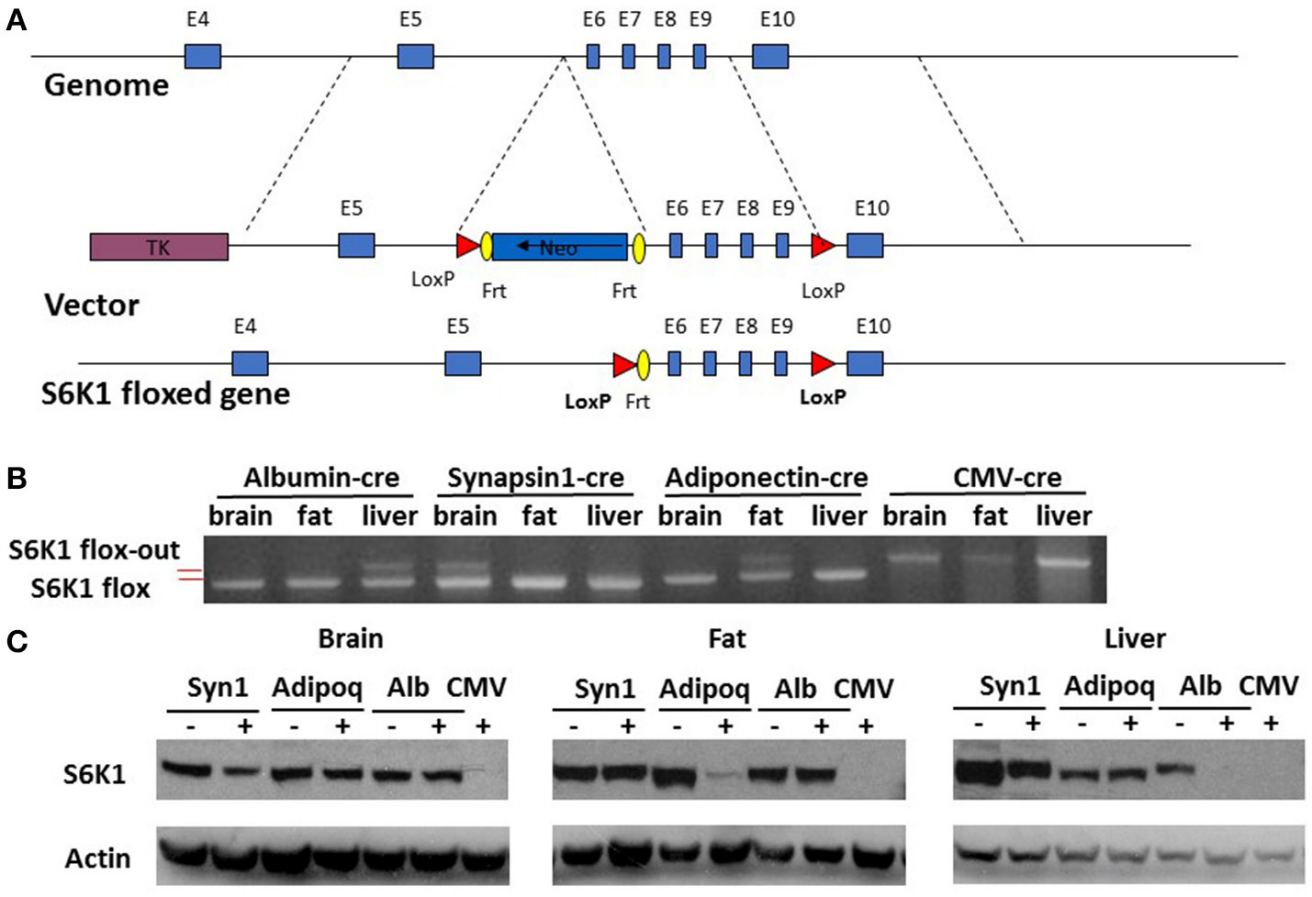
Generation of tissue specific S6K1 conditional KO mice. **(A)** Schematic targeting strategy to create tissue specific disruption of the S6K1 gene. **(B)** Genotyping PCR for S6K1 floxed alleles in brain, fat, and liver collected from floxed mice crossed with Albumin-cre, Synapsin1-cre, Adiponectin-cre, and CMV-cre mice. Upper bands indicate flox-out alleles. Lower bands indicate intact flox alleles. **(C)** Western blot with S6K1 and actin antibody in brain, fat, and liver, collected from floxed mice crossed with Synapsin1-cre (Syn1), Adiponectin-cre (Adipoq), Albumin-cre (Alb), and CMV-cre mice.

Mice were previously generated with a conditional allele of the S6K1 (*Rps6kb1*) gene with exon 6–9 flanked by loxP sites (Figure 1A) (McQuary et al., 2016). Homozygous S6K1 floxed mice (S6K1^fl/fl^) appeared normal, without obvious phenotypic abnormalities. We also previously generated a CMV-cre line expressing a transgene containing Cre recombinase under the transcriptional control of a human cytomegalovirus minimal promoter (McQuary et al., 2016). Deletion of exon 6–9 of S6K1 in the whole body by crossing S6K1^fl/fl^ mice with the CMV-cre line resulted in recombination in all tissues examined (Figure 1B and Supplemental Figure 1; McQuary et al., 2016). S6K1^fl/fl^ mice bred to mice expressing Cre recombinase directed by the promoter/regulatory regions of Adiponetcin (Adipoq-cre) (Eguchi et al., 2011), Synapsin I (Syn1-cre) (Zhu et al., 2001), or Albumin (Alb-cre) (Postic et al., 1999) had the S6K1 gene deleted specifically with gene recombination restricted in adipocytes (fat), neurons (brain), or hepatocytes (liver), respectively. In every case, intact S6K1 flox alleles were detected in tissues not expressing Cre recombinase (Figure 1B and Supplemetnal Figure 1). Western blotting revealed complete loss of S6K1 proteins in all tissues of CMV-cre; S6K1^fl/fl^ mice (Figure 1C). S6K1^fl/fl^ mice crossed with Syn1-cre, Adipoq-cre, and Alb-cre showed reduced S6K1 protein in brain, fat, and liver, respectively, compared to their littermates without the presence of Cre recombinase (Figure 1C). Considering the gene expression patterns of the promoters used, these results likely reflect the knock out of S6K1 in parenchymal cells but not in mesenchymal cells in each tissue analyzed. Thus, we successfully created mouse lines with disruption of S6K1 in the brain, fat, liver, or the whole body.

**Figure 2:**
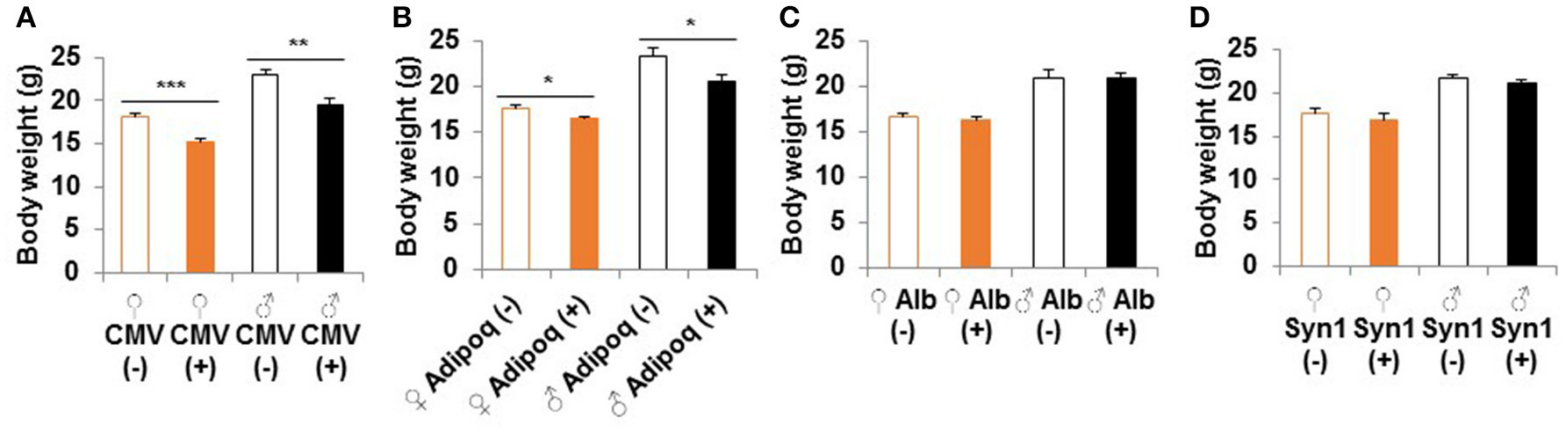
Body size of S6K1 conditional KO strains. Body weight at 2 months of age of **(A)** whole body (CMV, *n* = 8–10 for female and *n* = 7–10 for male), **(B)** fat-specific (Adipoq, *n* = 6–7 for female and *n* = 4–6 for male), **(C)** liver-specific (Alb, *n* = 13–16 for female and *n* = 5–7 for male), and **(D)** brain-specific (Syn1, *n* = 7–8 for female and *n* = 8–11 for male) S6K1 conditional KO mice. Data are indicated as mean ± s.e.m. ^*^p < 0.05, ^**^p < 0.01, ^***^p < 0.001.

Homozygous whole body S6K1 KO by CMV-cre resulted in a reduced body weight compared to littermate heterozygous controls without Cre (S6K1^fl/Δ^) at 2 months of age (Figure 2A). This weight reduction is consistent with a prior report on conventional S6K1 KO mice (Shima et al., 1998). S6K1 deletion in the fat by Adipoq-cre also caused smaller body size in both male and female mice compared to littermate controls (Figure 2B). There was a significant reduction in size of brain, kidney, colon, and visceral white adipose tissue in the whole body knockout, whereas a significant decrease was only observed in visceral white fat in the fat-specific KO (Supplemental Figure 2). Liver or brain conditional disruption of S6K1 by Alb-cre or Syn1-cre did not result in a significant change in body weight compared with littermates without cre (Figures 2C,D). Consistent with the unaltered body size, none of the organs analyzed showed a significant change in size in the brain-specific KO (Supplemental Figure 3A). In the liver-specific S6K1 mice, no significant changes were observed in liver size or body fat mass ratio at either 2 months of age (Supplemental Figures 3B–D) or 12 months of age (Supplemental Figures 3E–G).

NKO mice show a progressive neurodegenerative phenotype that recapitulates the clinical features of Leigh Syndrome and die at a median age of ~2 months (Kruse et al., 2008; Quintana et al., 2010). when NKO mice were bred to the S6K1 floxed mouse strain, we detected no significant difference in survival among S6k1^fl/fl^, S6K1^fl/+^, and S6K1^+/+^ alleles in the NKO background (Supplemental Figure 4). These lifespan data were comparable to our historical data for the NKO strain (Supplemental Figure 4).

**Figure 3:**
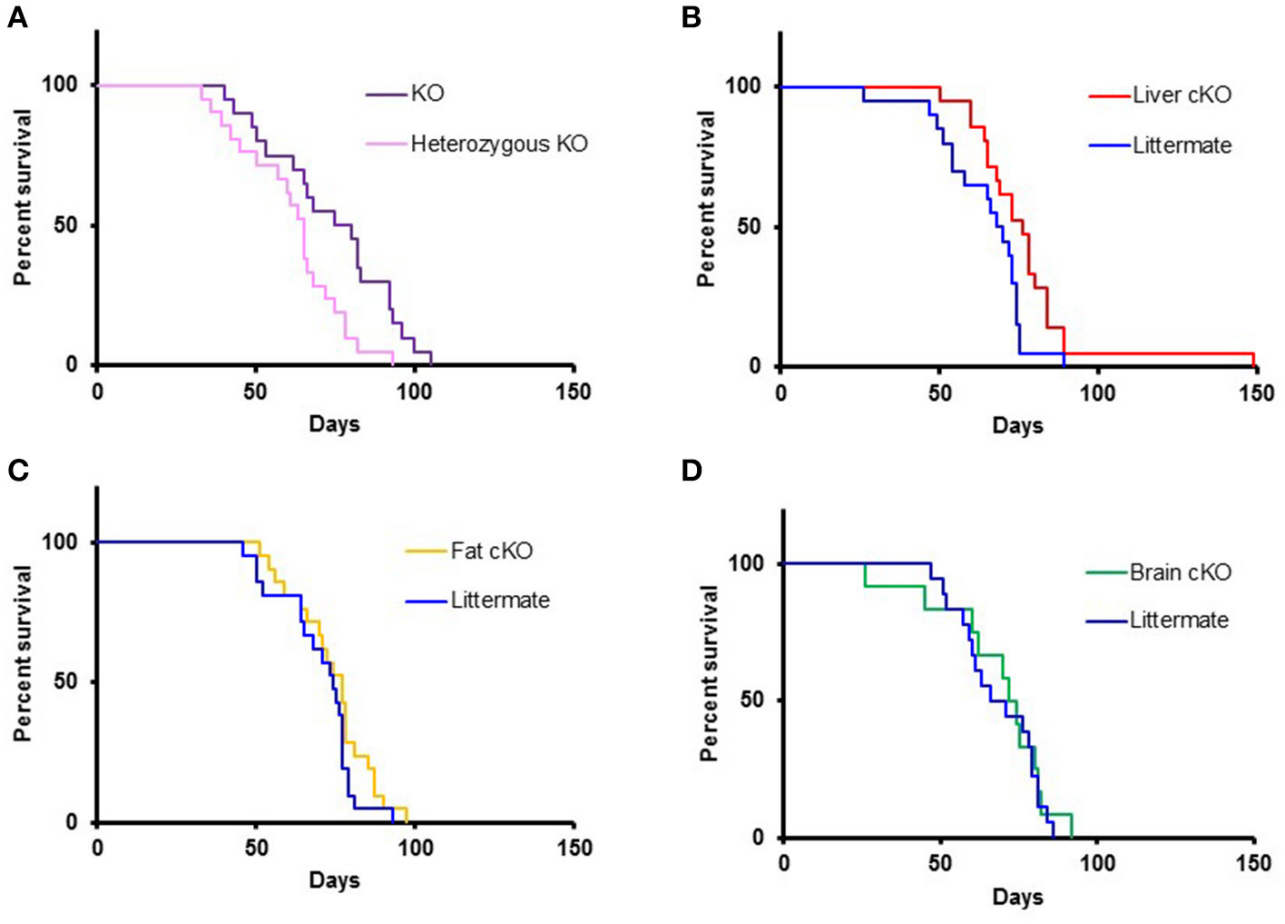
Lifespan of S6K1 conditional KO strains in the Ndufs4 ^−/−^ background. **(A)** Lifespan of homozygous S6K1 flox-out (whole body S6K1 KO, *n* = 20, 6 female and 14 male) and their littermate heterozygous S6K1 flox-out (*n* = 21, 12 female and 9 male) by CMV-cre. *P* < 0.05. **(B)** Lifespan of Albumin-cre; S6K1^fl/fl^; *Ndufs4*^−/−^ (Liver S6K1 cKO, *n* = 21, 9 female and 12 male) and their littermates without the cre gene (*n* = 20, 10 female and 10 male). *p* < 0.05. **(C)** Lifespan of Adiponectin-cre; S6K1^fl/fl^; *Ndufs4*^−/−^ (Fat S6K1 cKO, *n* = 21, 15 female and 6 male) and their littermates without the cre gene (*n* = 21, 8 female and 13 male). **(D)** Lifespan of Synapsin1-cre; S6K1^fl/fl^; *Ndufs4*^−/−^ (Brain S6K1 cKO, *n* = 12, 5 female and 7 male) and their littermates without the cre gene (*n* = 18, 11 female and 7 male).

Homozygous whole body disruption of S6K1 in the NKO background resulted in an increase of the median lifespan by 16% compared to the lifespan of littermate controls (Figure 3A, *p* = 0.01). A similar increase in survival was observed when disruption of S6K1 was restricted to the liver (Figure 3B, *p* = 0.01). In contrast, disruption of S6K1 in either fat or brain had no effect on survival of NKO mice (Figures 3C,D).

**Figure 4:**
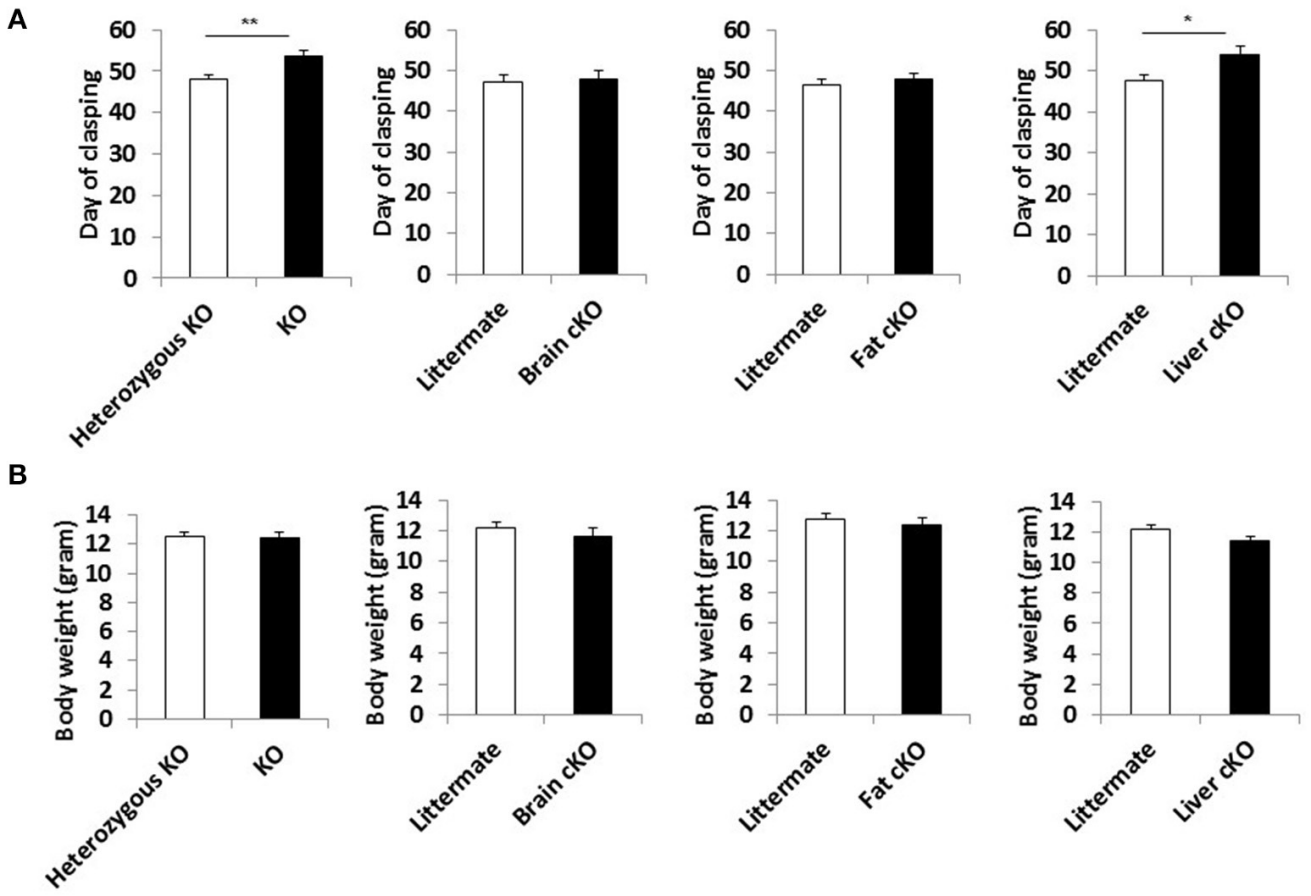
Phenotype of S6K1 conditional KO strains in the Ndufs4 ^−/−^ background. **(A)** Maximum body weight each mouse reached during the lifespan analysis for whole body S6K1 KO (KO by CMV-cre) and tissue-specific S6K1 cKOs (brain cKO by Syn1-cre, fat cKO by Adipoq-cre, and liver cKO by Alb-cre) in the Ndufs4 KO strains. **(B)** Age at which animals first show the clasping phenotype as an indicator of neurological dysfunction. Data are indicated as mean ± s.e.m. ^*^p < 0.05, ^**^p < 0.01.

We measured the incidence of forelimb clasping, an easily scored, noninvasive neurological phenotype that correlates with neurodegeneration in the NKO mice (Johnson et al., 2013b). The time at which the clasping phenotype appeared was delayed by the whole body S6K1 deletion and liver-specific deletion (Figure 4A), showing that neuronal dysfunction was mitigated in these lines along with the lifespan extension. None of the tissue specific or whole body S6K1 deletions significantly affected the body weight of NKO mice (Figure 4B and Supplemental Figure 5).

## Discussion

In this study, we found that inactivation of the mTOR substrate S6K1 in the whole body modestly enhances survival and delays the onset of a characteristic neurological symptom in the NKO mouse model of severe mitochondrial disease resulting from Complex I deficiency. Similar effects were observed when disruption of S6K1 was restricted to liver, while the disruption of S6K1 in brain or fat had no effect on disease progression. These observations are consistent with the model that S6K1 acts downstream of mTOR to mediate mitochondrial disease in this mouse model and that the liver is the primary tissue of action for S6K1 in this context.

The positive impact of S6K1 disruption in liver but not in brain of NKO mice is somewhat counterintuitive, as neurodegeneration is the primary cause of morbidity and mortality in both the NKO mouse as well as in patients with Leigh Syndrome. It is important to note that the Synapsin I cre used here to disrupt S6K1 in the brain is a neuron-specific promoter, so it remains possible that S6K1 activity in other brain cell types, such as glia, could be important for disease progression. Nonetheless, the ability of hepatic cell S6K1 knockout to enhance survival and delay neurological symptoms clearly indicates a role for mTOR signaling in liver in this disease. This is consistent with our prior data indicating that rapamycin treatment in NKO mice results in substantial changes in protein expression and metabolite levels in the liver, including evidence for increased triglyceride levels and elevated amino acid catabolism (Johnson et al., 2015b).

Although, whole body or liver-specific S6K1 disruption was not as effective as daily injection of 8 mg/kg rapamycin at enhancing health and survival in the NKO mice, the effects observed here were comparable to lower dose regimens of rapamycin treatment in this background (Supplemental Figure 6; Johnson et al., 2013b, 2015b). This suggests that the effects of rapamycin can be explained in part by reducing S6K1 activity, but that additional downstream effects of mTORC1 inhibition also likely contribute to delayed mitochondrial disease progression in NKO mice at higher doses of rapamycin. Other outputs of mTORC1 signaling, such as 4EBP1 and autophagy (Johnson et al., 2013a), which are also associated with normative aging, are likely candidates. Alternatively, mTORC2 signaling may play a role in mitochondrial disease, since chronic mTORC1 inhibition by rapamycin can inhibit mTORC2 activity (Sarbassov et al., 2006). It is also possible that S6K1 disruption is inducing changes in the signaling network through its regulation of the insulin/IGF-1 signaling network (Johnson et al., 2013a) or additional targets, or that S6K1 disruption is alleviating mitochondrial disease in these animals by a mechanism distinct from rapamycin.

While this study focused on the impact of S6K1 and mTOR signaling in severe mitochondrial disease, these results may have relevance to normative aging as well. Prior studies of mTORC1 signaling and S6K1 in the context of normative aging have largely focused on whole body genetic disruptions (Selman et al., 2009; Lamming et al., 2012; Wu et al., 2013) or systemic treatment with rapamycin (Harrison et al., 2009; Miller et al., 2011, 2014; Wilkinson et al., 2012; Neff et al., 2013; Fok et al., 2014; Popovich et al., 2014; Arriola Apelo et al., 2016). Our results indicate that liver specific disruption of S6K1 can result in delayed pathology in the brain during mitochondrial disease and suggest the potential importance of cell and tissue non-autonomous systemic effects during normative aging. This could be particularly relevant for effects of rapamycin on cognitive aging and dementia, as numerous studies have shown that rapamycin and mTOR inhibition can delay cognitive decline during normative aging (Halloran et al., 2012; Majumder et al., 2012) as well as prevent or reverse Alzheimer's disease progression (Spilman et al., 2010; Majumder et al., 2011; Lin et al., 2013, 2017; Ozcelik et al., 2013; Caccamo et al., 2014; Jiang et al., 2014; Siman et al., 2015) in mouse models. It would be informative to understand the impact of tissue specific depletion or disruption of mTORC1 components or S6K1 on healthy longevity, cognition, and Alzheimer's disease in mice. Likewise, local delivery of rapamycin or other mTOR inhibitors could have distinct effects from systemic treatments, both in terms of overall improvements in healthspan and lifespan, as well as lowering risks for adverse side effects.

In summary, we show here that whole body disruption or liver specific disruption of the mTOR substrate S6K1 can increase survival and delay disease symptoms in a mouse model of severe mitochondrial disease. These observations are consistent with the idea that reduced S6K1 activity accounts for at least part of the beneficial effect of rapamycin in this context, and suggests that inhibition of S6K1 may be a viable therapeutic strategy for treating mitochondrial disease. Importantly, these data also suggest that inhibition of mTORC1 and S6K1 in the liver is critically important for delaying mitochondrial disease in the brain, perhaps through a change in systemic metabolism that attenuates neuroinflammation and neurodegeneration.

## Author contributions

MK conceptualized the project. TI and MK designed the experiments, analyzed the data, and wrote the manuscript. JM and WL generated and distributed S6K1 floxed mice and CMV-cre recombinase strain. TI, CL, JK, QN, HH, DK, JP, JT, YL, TN, SK, NL, SM, WW, AB, and JA bred mice according to genotyping PCR analyses. TI, CL, HH, DK, JP, JT, YL, TN, NL, and SK analyzed body weights, monitored health conditions including the clasping phenotype, and performed the lifespan analysis. HH, JO, and VP performed western blot. TI, CL, JK, QN, HH, and WW performed body composition analysis. MK and MS supervised the project and provided essential resources for project completion.

### Conflict of interest statement

The authors declare that the research was conducted in the absence of any commercial or financial relationships that could be construed as a potential conflict of interest.

## References

[B1] Arif A. Terenzi F. Potdar A. A. Jia J. Sacks J. China A.. (2017). EPRS is a critical mTORC1-S6K1 effector that influences adiposity in mice. Nature 542, 357–361. 10.1038/nature21380 28178239 PMC5480610

[B2] Arriola Apelo S. I. Pumper C. P. Baar E. L. Cummings N. E. Lamming D. W. (2016). Intermittent administration of rapamycin extends the life span of female C57BL/6J mice. J. Gerontol. A Biol. Sci. Med. Sci. 71, 876–881. 10.1093/gerona/glw064 27091134 PMC4906329

[B3] Bitto A. Ito T. K. Pineda V. V. LeTexier N. J. Huang H. Z. Sutlief E.. (2016). Transient rapamycin treatment can increase lifespan and healthspan in middle-aged mice. Elife 5:e16351. 10.7554/eLife.16351 27549339 PMC4996648

[B4] Blagosklonny M. V. (2006). Aging and immortality: quasi-programmed senescence and its pharmacologic inhibition. Cell Cycle 5, 2087–2102. 10.4161/cc.5.18.3288 17012837

[B5] Caccamo A. De Pinto V. Messina A. Branca C. Oddo S. (2014). Genetic reduction of mammalian target of rapamycin ameliorates Alzheimer's disease-like cognitive and pathological deficits by restoring hippocampal gene expression signature. J. Neurosci. 34, 7988–7998. 10.1523/JNEUROSCI.0777-14.2014 24899720 PMC4044255

[B6] Eguchi J. Wang X. Yu S. Kershaw E. E. Chiu P. C. Dushay J.. (2011). Transcriptional control of adipose lipid handling by IRF4. Cell Metab. 13, 249–259. 10.1016/j.cmet.2011.02.005 21356515 PMC3063358

[B7] Fok W. C. Chen Y. Bokov A. Zhang Y. Salmon A. B. Diaz V.. (2014). Mice fed rapamycin have an increase in lifespan associated with major changes in the liver transcriptome. PLoS ONE 9:e83988. 10.1371/journal.pone.0083988 24409289 PMC3883653

[B8] Halloran J. Hussong S. A. Burbank R. Podlutskaya N. Fischer K. E. Sloane L. B.. (2012). Chronic inhibition of mammalian target of rapamycin by rapamycin modulates cognitive and non-cognitive components of behavior throughout lifespan in mice. Neuroscience 223, 102–113. 10.1016/j.neuroscience.2012.06.054 22750207 PMC3454865

[B9] Harrison D. E. Strong R. Sharp Z. D. Nelson J. F. Astle C. M. Flurkey K.. (2009). Rapamycin fed late in life extends lifespan in genetically heterogeneous mice. Nature 460, 392–395. 10.1038/nature08221 19587680 PMC2786175

[B10] Jiang T. Yu J. T. Zhu X. C. Tan M. S. Wang H. F. Cao L.. (2014). Temsirolimus promotes autophagic clearance of amyloid-beta and provides protective effects in cellular and animal models of Alzheimer's disease. Pharmacol. Res. 81, 54–63. 10.1016/j.phrs.2014.02.008 24602800

[B11] Johnson S. C. Rabinovitch P. S. Kaeberlein M. (2013a). mTOR is a key modulator of ageing and age-related disease. Nature 493, 338–345. 10.1038/nature11861 23325216 PMC3687363

[B12] Johnson S. C. Sangesland M. Kaeberlein M. Rabinovitch P. S. (2015a). Modulating mTOR in aging and health. Interdiscip. Top. Gerontol. 40, 107–127. 10.1159/000364974 25341517

[B13] Johnson S. C. Yanos M. E. Bitto A. Castanza A. Gagnidze A. Gonzalez B.. (2015b). Dose-dependent effects of mTOR inhibition on weight and mitochondrial disease in mice. Front. Genet. 6:247. 10.3389/fgene.2015.00247 26257774 PMC4510413

[B14] Johnson S. C. Yanos M. E. Kayser E. B. Quintana A. Sangesland M. Castanza A.. (2013b). mTOR inhibition alleviates mitochondrial disease in a mouse model of leigh syndrome. Science 342, 1524–1528. 10.1126/science.1244360 24231806 PMC4055856

[B15] Kapahi P. Zid B. M. Harper T. Koslover D. Sapin V. Benzer S. (2004). Regulation of lifespan in Drosophila by modulation of genes in the TOR signaling pathway. Curr. Biol. 14, 885–890. 10.1016/j.cub.2004.03.059 15186745 PMC2754830

[B16] Kauppila T. E. Kauppila J. H. Larsson N. G. (2017). Mammalian mitochondria and aging: an update. Cell Metab. 25, 57–71. 10.1016/j.cmet.2016.09.017 28094012

[B17] Kim S. H. Scott S. A. Bennett M. J. Carson R. P. Fessel J. Brown H. A.. (2013). Multi-organ abnormalities and mTORC1 activation in zebrafish model of multiple acyl-CoA dehydrogenase deficiency. PLoS Genet. 9:e1003563. 10.1371/journal.pgen.1003563 23785301 PMC3681725

[B18] Kruse S. E. Watt W. C. Marcinek D. J. Kapur R. P. Schenkman K. A. Palmiter R. D. (2008). Mice with mitochondrial complex I deficiency develop a fatal encephalomyopathy. Cell Metab. 7, 312–320. 10.1016/j.cmet.2008.02.004 18396137 PMC2593686

[B19] Lamming D. W. Ye L. Katajisto P. Goncalves M. D. Saitoh M. Stevens D. M.. (2012). Rapamycin-induced insulin resistance is mediated by mTORC2 loss and uncoupled from longevity. Science 335, 1638–1643. 10.1126/science.1215135 22461615 PMC3324089

[B20] Laplante M. Sabatini D. M. (2013). Regulation of mTORC1 and its impact on gene expression at a glance. J. Cell Sci. 126(Pt 8), 1713–1719. 10.1242/jcs.125773 23641065 PMC3678406

[B21] Lin A. L. Jahrling J. B. Zhang W. DeRosa N. Bakshi V. Romero P.. (2017). Rapamycin rescues vascular, metabolic and learning deficits in apolipoprotein E4 transgenic mice with pre-symptomatic Alzheimer's disease. J. Cereb. Blood Flow Metab. 37, 217–226. 10.1177/0271678X15621575 26721390 PMC5167110

[B22] Lin A. L. Zheng W. Halloran J. J. Burbank R. R. Hussong S. A. Hart M. J.. (2013). Chronic rapamycin restores brain vascular integrity and function through NO synthase activation and improves memory in symptomatic mice modeling Alzheimer's disease. J. Cereb. Blood Flow Metab. 33, 1412–1421. 10.1038/jcbfm.2013.82 23801246 PMC3764385

[B23] Lombes A. Bonilla E. Dimauro S. (1989). Mitochondrial encephalomyopathies. Rev. Neurol. 145, 671–689. 2682927

[B24] Lopez-Otin C. Blasco M. A. Partridge L. Serrano M. Kroemer G. (2013). The hallmarks of aging. Cell 153, 1194–1217. 10.1016/j.cell.2013.05.039 23746838 PMC3836174

[B25] Majumder S. Caccamo A. Medina D. X. Benavides A. D. Javors M. A. Kraig E.. (2012). Lifelong rapamycin administration ameliorates age-dependent cognitive deficits by reducing IL-1beta and enhancing NMDA signaling. Aging Cell 11, 326–335. 10.1111/j.1474-9726.2011.00791.x 22212527 PMC3306461

[B26] Majumder S. Richardson A. Strong R. Oddo S. (2011). Inducing autophagy by rapamycin before, but not after, the formation of plaques and tangles ameliorates cognitive deficits. PLoS ONE 6:e25416. 10.1371/journal.pone.0025416 21980451 PMC3182203

[B27] McQuary P. R. Liao C. Y. Chang J. T. Kumsta C. She X. Davis A.. (2016). *C elegans* S6K mutants require a creatine-kinase-like effector for lifespan extension. Cell Rep. 14, 2059–2067. 10.1016/j.celrep.2016.02.012 26923601 PMC4823261

[B28] Miller R. A. Harrison D. E. Astle C. M. Baur J. A. Boyd A. R. de Cabo R.. (2011). Rapamycin, but not resveratrol or simvastatin, extends life span of genetically heterogeneous mice. J. Gerontol. A Biol. Sci. Med. Sci. 66, 191–201. 10.1093/gerona/glq178 20974732 PMC3021372

[B29] Miller R. A. Harrison D. E. Astle C. M. Fernandez E. Flurkey K. Han M.. (2014). Rapamycin-mediated lifespan increase in mice is dose and sex dependent and metabolically distinct from dietary restriction. Aging Cell 13, 468–477. 10.1111/acel.12194 24341993 PMC4032600

[B30] Neff F. Flores-Dominguez D. Ryan D. P. Horsch M. Schroder S. Adler T.. (2013). Rapamycin extends murine lifespan but has limited effects on aging. J. Clin. Invest. 123, 3272–3291. 10.1172/JCI67674 23863708 PMC3726163

[B31] Ortigoza-Escobar J. D. Oyarzabal A. Montero R. Artuch R. Jou C. Jimenez C.. (2016). Ndufs4 related leigh syndrome: a case report and review of the literature. Mitochondrion 28, 73–78. 10.1016/j.mito.2016.04.001 27079373

[B32] Ozcelik S. Fraser G. Castets P. Schaeffer V. Skachokova Z. Breu K.. (2013). Rapamycin attenuates the progression of tau pathology in P301S tau transgenic mice. PLoS ONE 8:e62459. 10.1371/journal.pone.0062459 23667480 PMC3646815

[B33] Payne B. A. Chinnery P. F. (2015). Mitochondrial dysfunction in aging: much progress but many unresolved questions. Biochim. Biophys. Acta 1847, 1347–1353. 10.1016/j.bbabio.2015.05.022 26050973 PMC4580208

[B34] Peng M. Ostrovsky J. Kwon Y. J. Polyak E. Licata J. Tsukikawa M.. (2015). Inhibiting cytosolic translation and autophagy improves health in mitochondrial disease. Hum. Mol. Genet. 24, 4829–4847. 10.1093/hmg/ddv207 26041819 PMC4527487

[B35] Popovich I. G. Anisimov V. N. Zabezhinski M. A. Semenchenko A. V. Tyndyk M. L. Yurova M. N.. (2014). Lifespan extension and cancer prevention in HER-2/neu transgenic mice treated with low intermittent doses of rapamycin. Cancer Biol. Ther. 15, 586–592. 10.4161/cbt.28164 24556924 PMC4026081

[B36] Postic C. Shiota M. Niswender K. D. Jetton T. L. Chen Y. Moates J. M.. (1999). Dual roles for glucokinase in glucose homeostasis as determined by liver and pancreatic beta cell-specific gene knock-outs using Cre recombinase. J. Biol. Chem. 274, 305–315. 10.1074/jbc.274.1.305 9867845

[B37] Quintana A. Kruse S. E. Kapur R. P. Sanz E. Palmiter R. D. (2010). Complex I deficiency due to loss of Ndufs4 in the brain results in progressive encephalopathy resembling Leigh syndrome. Proc. Natl. Acad. Sci. U.S.A. 107, 10996–11001. 10.1073/pnas.1006214107 20534480 PMC2890717

[B38] Rose G. Santoro A. Salvioli S. (2016). Mitochondria and mitochondria-induced signalling molecules as longevity determinants. Mech. Ageing Dev. [Epub ahead of print]. 10.1016/j.mad.2016.12.002 27964991

[B39] Sarbassov D. D. Ali S. M. Sengupta S. Sheen J. H. Hsu P. P. Bagley A. F.. (2006). Prolonged rapamycin treatment inhibits mTORC2 assembly and Akt/PKB. Mol. Cell 22, 159–168. 10.1016/j.molcel.2006.03.029 16603397

[B40] Schleit J. Johnson S. C. Bennett C. F. Simko M. Trongtham N. Castanza A.. (2013). Molecular mechanisms underlying genotype-dependent responses to dietary restriction. Aging Cell 12, 1050–1061. 10.1111/acel.12130 23837470 PMC3838465

[B41] Sebastian D. Palacin M. Zorzano A. (2017). Mitochondrial dynamics: coupling mitochondrial fitness with healthy aging. Trends Mol. Med. 23, 201–215. 10.1016/j.molmed.2017.01.003 28188102

[B42] Selman C. Tullet J. M. Wieser D. Irvine E. Lingard S. J. Choudhury A. I.. (2009). Ribosomal protein S6 kinase 1 signaling regulates mammalian life span. Science 326, 140–144. 10.1126/science.1177221 19797661 PMC4954603

[B43] Shima H. Pende M. Chen Y. Fumagalli S. Thomas G. Kozma S. C. (1998). Disruption of the p70(s6k)/p85(s6k) gene reveals a small mouse phenotype and a new functional S6 kinase. EMBO J. 17, 6649–6659. 10.1093/emboj/17.22.6649 9822608 PMC1171010

[B44] Siman R. Cocca R. Dong Y. (2015). The mTOR inhibitor rapamycin mitigates perforant pathway neurodegeneration and synapse loss in a mouse model of early-stage alzheimer-type tauopathy. PLoS ONE 10:e0142340. 10.1371/journal.pone.0142340 26540269 PMC4634963

[B45] Spilman P. Podlutskaya N. Hart M. J. Debnath J. Gorostiza O. Bredesen D.. (2010). Inhibition of mTOR by rapamycin abolishes cognitive deficits and reduces amyloid-beta levels in a mouse model of Alzheimer's disease. PLoS ONE 5:e9979. 10.1371/journal.pone.0009979 20376313 PMC2848616

[B46] Wang A. Mouser J. Pitt J. Promislow D. Kaeberlein M. (2016). Rapamycin enhances survival in a Drosophila model of mitochondrial disease. Oncotarget 7, 80131–80139. 10.18632/oncotarget.12560 27741510 PMC5348310

[B47] Wilkinson J. E. Burmeister L. Brooks S. V. Chan C. C. Friedline S. Harrison D. E.. (2012). Rapamycin slows aging in mice. Aging Cell 11, 675–682. 10.1111/j.1474-9726.2012.00832.x 22587563 PMC3434687

[B48] Wu J. J. Liu J. Chen E. B. Wang J. J. Cao L. Narayan N.. (2013). Increased mammalian lifespan and a segmental and tissue-specific slowing of aging after genetic reduction of mTOR expression. Cell Rep. 4, 913–920. 10.1016/j.celrep.2013.07.030 23994476 PMC3784301

[B49] Zheng X. Boyer L. Jin M. Kim Y. Fan W. Bardy C.. (2016). Alleviation of neuronal energy deficiency by mTOR inhibition as a treatment for mitochondria-related neurodegeneration. Elife 5:e13378. 10.7554/eLife.13378 27008180 PMC4846388

[B50] Zhu Y. Romero M. I. Ghosh P. Ye Z. Charnay P. Rushing E. J.. (2001). Ablation of NF1 function in neurons induces abnormal development of cerebral cortex and reactive gliosis in the brain. Genes Dev. 15, 859–876. 10.1101/gad.862101 11297510 PMC312666

