# Corrigendum: Hepatic S6K1 Partially Regulates Lifespan of Mice with Mitochondrial Complex I Deficiency

**DOI:** 10.3389/fgene.2017.00221

**Published:** 2017-12-22

**Authors:** Takashi K. Ito, Chenhao Lu, Jacob Khan, Quy Nguyen, Heather Z. Huang, Dayae Kim, James J. Phillips, Jo Tan, Yenna Lee, Tuyet Nguyen, Samy Khessib, Natalie Lim, Surapat Mekvanich, Joshua Oh, Victor V. Pineda, Weirong Wang, Alessandro Bitto, Jonathan Y. An, John F. Morton, Mitsutoshi Setou, Warren C. Ladiges, Matt Kaeberlein

**Affiliations:** ^1^Department of Pathology, University of Washington, Seattle, WA, United States; ^2^Department of Cellular and Molecular Anatomy, Hamamatsu University School of Medicine, Hamamatsu, Japan; ^3^International Mass Imaging Center, Hamamatsu University School of Medicine, Hamamatsu, Japan; ^4^Research Institute of Atherosclerotic Disease, Xi'an Jiaotong University Cardiovascular Research Center, Xi'an, China; ^5^Laboratory Animal Center, Xi'an Jiaotong University Health Science Center, Xi'an, China; ^6^Department of Comparative Medicine, University of Washington, Seattle, WA, United States

**Keywords:** S6K1, mTORC1, liver, lifespan, mitochondrial disease

In the original article, we neglected to include relevant animal protocols belonging to Hamamatsu University School of Medicine (H28-068 and H29-083). A correction has been made to Materials and Methods, Animals, the second paragraph. All care of experimental animals was in accordance with the institutional guidelines of University of Washington and Hamamatsu University School of Medicine, and experiments were performed as approved by the Institutional Animal Care and Use Committees at the University of Washington (protocol #4359-03) and Hamamatsu University School of Medicine (protocols H28-068 and H29-083). The authors apologize for this error and state that this does not change the scientific conclusions of the article in any way.

The original article has been updated.

## Conflict of interest statement

The authors declare that the research was conducted in the absence of any commercial or financial relationships that could be construed as a potential conflict of interest.

