# Supplementary figures and images for: Hepatic S6K1 Partially Regulates Lifespan of Mice with Mitochondrial Complex I Deficiency
Source: Front Genet. 2017 Sep 1;8:113. doi: 10.3389/fgene.2017.00113 (PMC5585733; doi:10.3389/fgene.2017.00113)

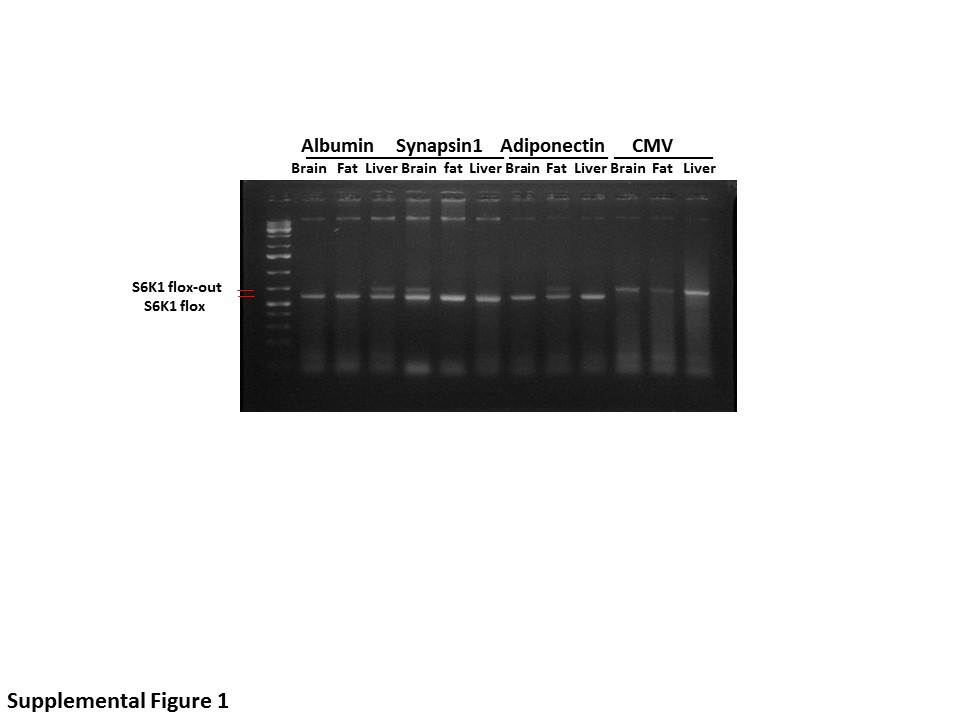

Supplement: Supplementary file 1 [file Image1.JPEG]

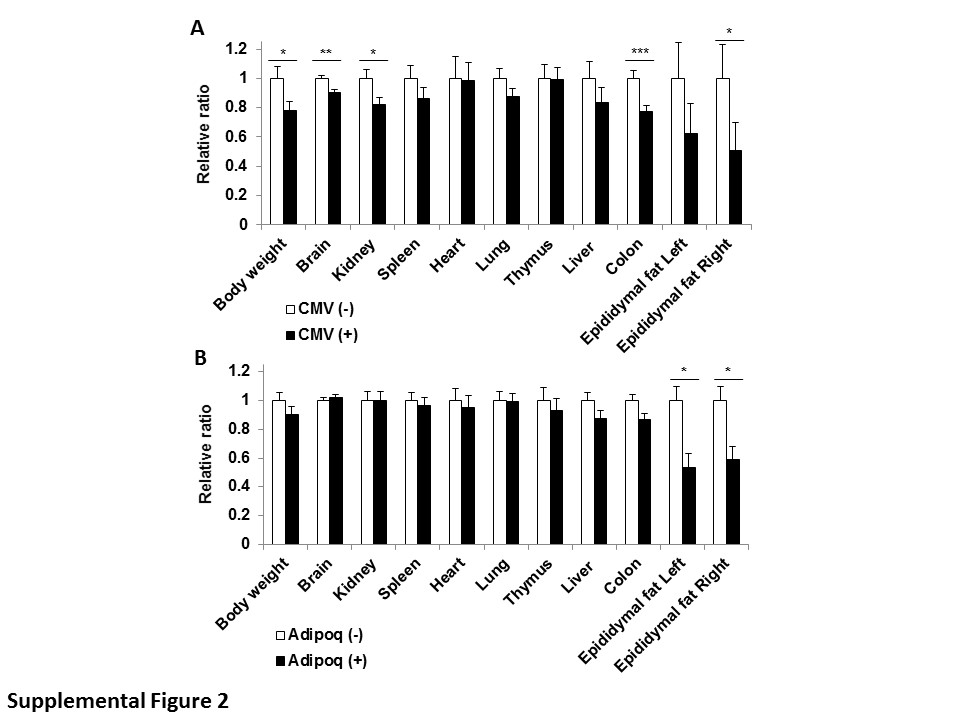

Supplement: Supplementary file 2 [file Image2.JPEG]

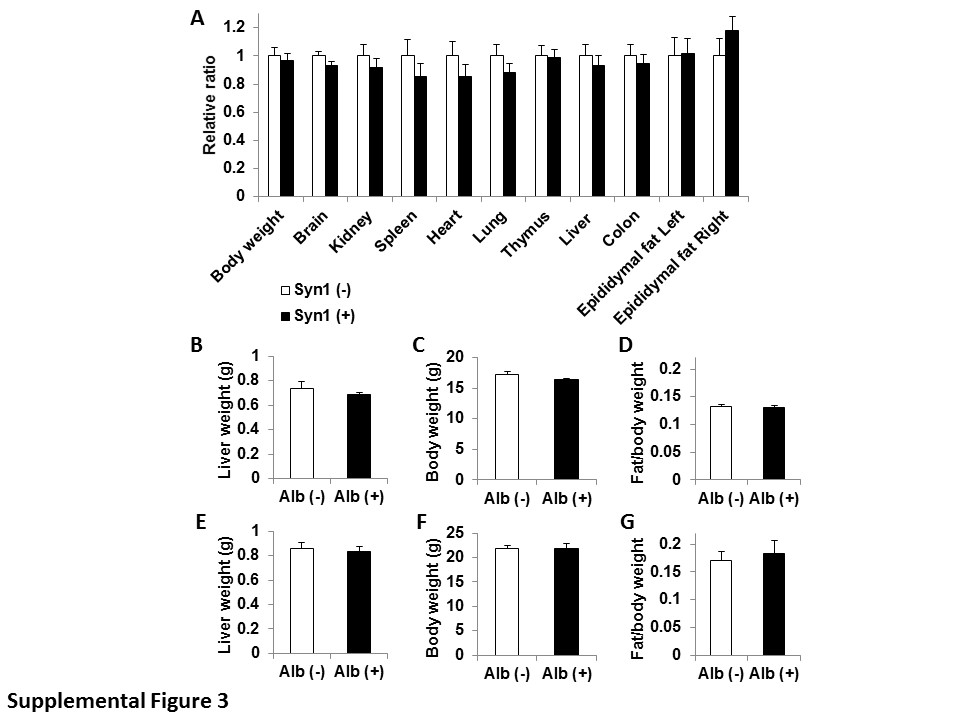

Supplement: Supplementary file 3 [file Image3.JPEG]

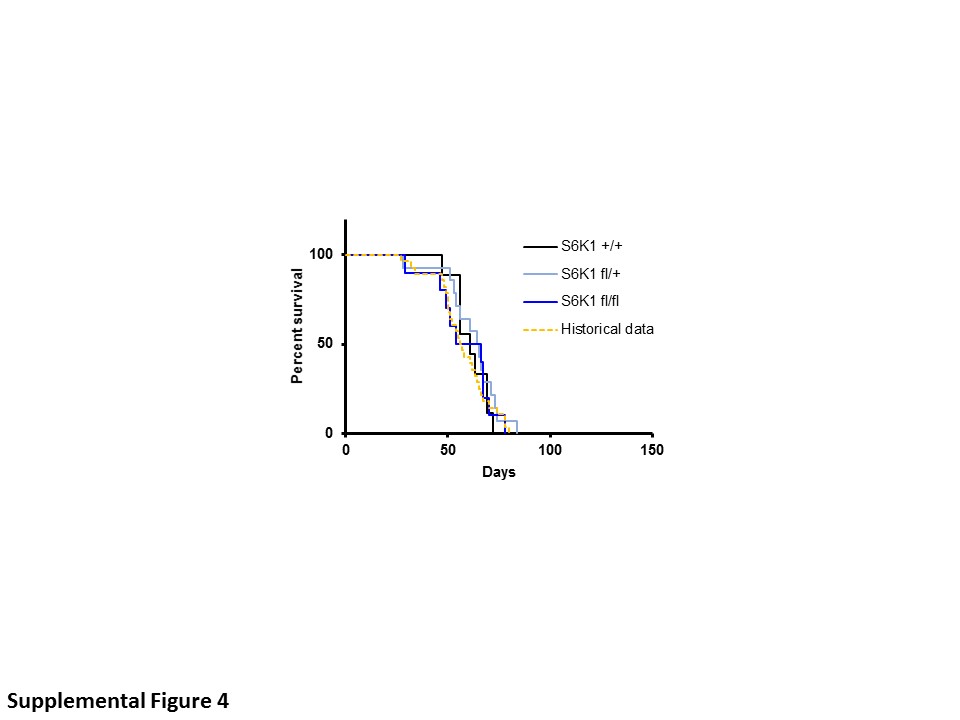

Supplement: Supplementary file 4 [file Image4.JPEG]

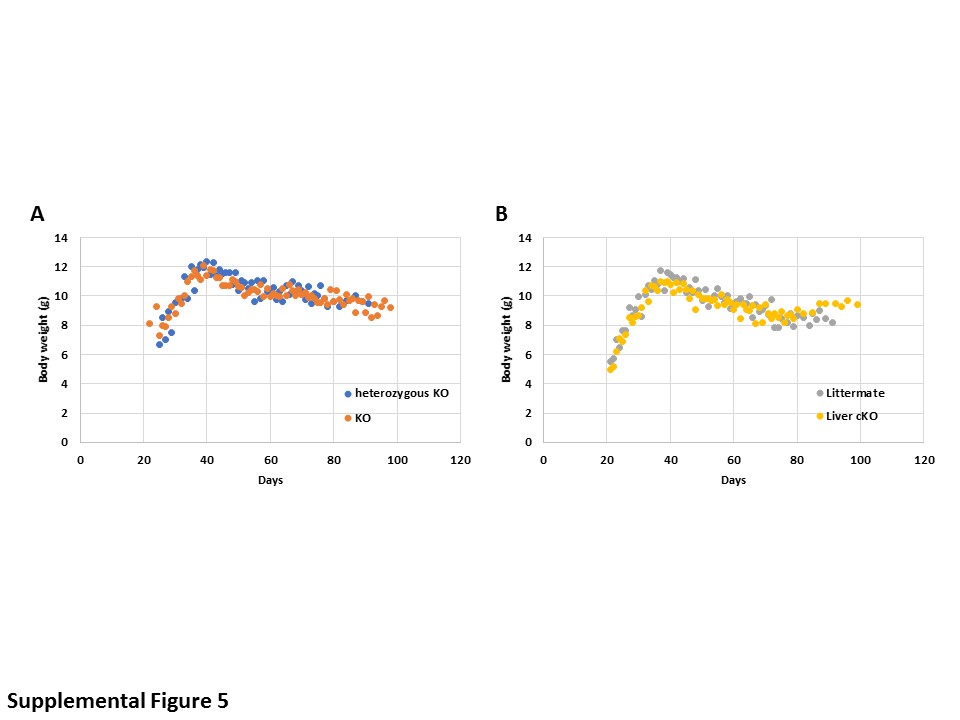

Supplement: Supplementary file 5 [file Image5.JPEG]

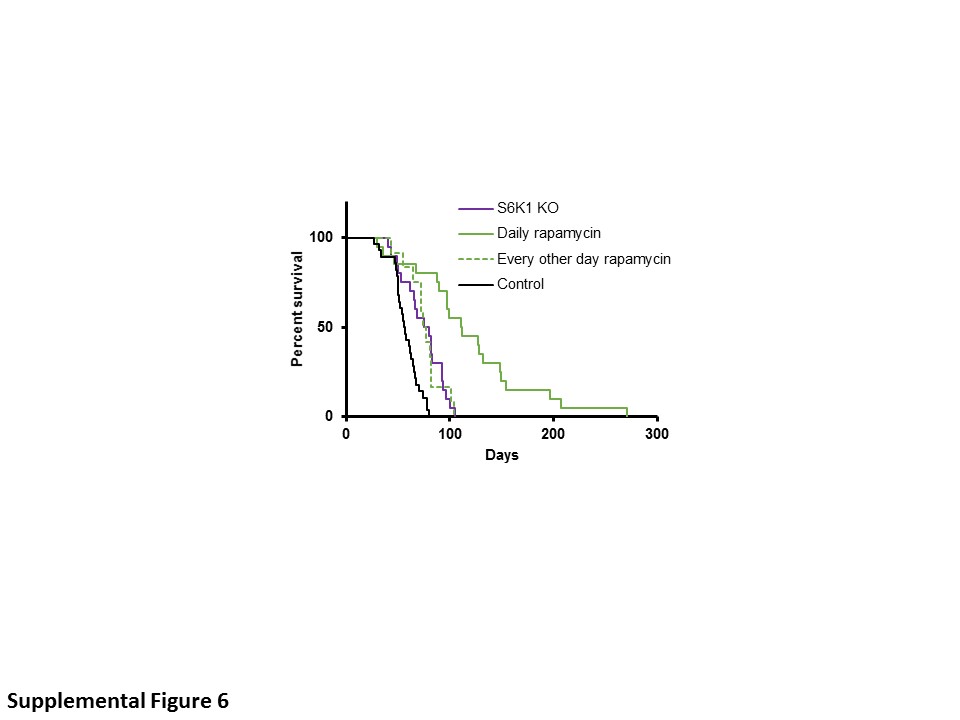

Supplement: Supplementary file 6 [file Image6.JPEG]
