# Supplementary material for: Hepatic S6K1 Partially Regulates Lifespan of Mice with Mitochondrial Complex I Deficiency
Source: Front Genet. 2017 Sep 1;8:113. doi: 10.3389/fgene.2017.00113 (PMC5585733; doi:10.3389/fgene.2017.00113)
Supplement: Supplementary file 7 [file Presentation1.PDF]

Supplemental figure legends

Supplemental Figure 1 | Genotyping PCR of tissue specific S6K1 conditional KO mice.

Image of the whole gel for genotyping PCR for S6K1 floxed alleles in brain, fat, and liver collected from floxed mice crossed with Albumin-cre, Synapsin1-cre, Adiponectin-cre, and CMV-cre mice. Upper bands indicate flox-out alleles. Lower bands indicate intact flox alleles. The sizes of PCR products are designed to be 480bp for WT (not detected), 580 bp for flox, and 656 bp for flox-out alleles.

Supplemental Figure 2 | Body composition of whole body and adipose-specific S6K1 conditional KO mice.

Tissue weights at 2 months of age of (A) whole body (CMV, n = 5, 3 female and 2 male for CMV(-), n = 7, 4 female and 3 male for CMV(+)) and (B) fat-specific (Adipoq, n = 6, 4 female and 2 male each for Adipoq(-) and Adipoq(+)) S6K1 conditional KO. Data are indicated as mean ± s.e.m. *p<0.05. **p<0.01. ***p<0.001.

Supplemental Figure 3 | Body composition of neuron-specific and liver-specific S6K1 conditional KO mice.

(A) Tissue weights at 2 months of age of brain-specific (Syn1, n = 6, 2 female and 4 male for Syn1(-), n = 9, 3 female and 6 male for Syn1(+)) S6K1 conditional KO. (B) Liver weight at 2 months of age of liver-specific (Alb, n = 3, female) S6K1 conditional KO. (C) Body weight and (D) body fat mass ratio at 2 months of age of liver-specific (Alb, female, n = 10 for Alb(-) and n = 8 for Alb(+)) S6K1 conditional KO. (E) Liver weight at 12 months of age of liver-specific (Alb, female, n = 4, all for Alb(-) and n = 5 for Alb(+)) S6K1 conditional KO. (F) Body weight and (G) body fat mass ratio at 12 months of age of liver-specific (Alb, female, n = 4 for Alb(-) and n = 5 for Alb(+)) S6K1 conditional KO. Data are indicated as mean ± s.e.m. *p<0.05. **p<0.01. ***p<0.001.

Supplemental Figure 4 | Survival of S6K1 floxed mice in the *Ndufs4*$^{-/-}$ background.

Survival of wildtype S6K1 (S6K1$^{+/+}$, n = 9, 5 female and 4 male), heterozygous S6K1 floxed (S6K1$^{fl/+}$, n = 14, 6 female and 8 male) and homozygous S6K1 floxed (S6K1$^{fl/fl}$, n = 10, 7 female and 3 male) mice in the NKO background produced from breeders crossed with the S6K1 floxed strain. Our historical lifespan data with the original

NKO (Johnson et al., Science 2013) is shown in a dotted line as a reference.

Supplemental Figure 5 | Body weight of curve of whole body and liver-specific S6K1 KO strains in the Ndufs4 -/- background.

Body weight during the course of survival analysis for (A) whole body S6K1 KO by CMV-cre and their littermate heterozygous S6K1 KO, and (B) liver S6K1 cKO by Alb-cre and their control littermates in the Ndufs4 KO background. Average data were shown for each day point when the data were available.

Supplemental Figure 6 | Survival of S6K1 KO compared with rapamycin treatments in the *Ndufs4*$^{-/-}$ background.

Survival of S6K1 KO in the NKO background shown in Figure 3A is plotted together with our historical lifespan data for rapamycin treatment regimens (either daily or every other day 8 mg/kg intraperitoneal injections) and control (Johnson et al., Science 2013) as a reference.
